# Regulation of the DNA damage response by ubiquitin conjugation

**DOI:** 10.3389/fgene.2015.00098

**Published:** 2015-03-10

**Authors:** Kerstin Brinkmann, Michael Schell, Thorsten Hoppe, Hamid Kashkar

**Affiliations:** ^1^Centre for Molecular Medicine Cologne and Institute for Medical Microbiology, Immunology and Hygiene, University Hospital of CologneCologne, Germany; ^2^Cologne Excellence Cluster on Cellular Stress Responses in Aging-Associated Diseases, University Hospital of CologneCologne, Germany; ^3^Institute for Genetics, University of CologneCologne, Germany

**Keywords:** DNA damage, apoptosis, ubiquitylation, genotoxic anti-cancer therapy, p53, Bcl-2

## Abstract

In response to DNA damage, cells activate a highly conserved and complex kinase-based signaling network, commonly referred to as the DNA damage response (DDR), to safeguard genomic integrity. The DDR consists of a set of tightly regulated events, including detection of DNA damage, accumulation of DNA repair factors at the site of damage, and finally physical repair of the lesion. Upon overwhelming damage the DDR provokes detrimental cellular actions by involving the apoptotic machinery and inducing a coordinated demise of the damaged cells (DNA damage-induced apoptosis, DDIA). These diverse actions involve transcriptional activation of several genes that govern the DDR. Moreover, recent observations highlighted the role of ubiquitylation in orchestrating the DDR, providing a dynamic cellular regulatory circuit helping to guarantee genomic stability and cellular homeostasis (Popovic et al., 2014). One of the hallmarks of human cancer is genomic instability (Hanahan and Weinberg, 2011). Not surprisingly, deregulation of the DDR can lead to human diseases, including cancer, and can induce resistance to genotoxic anti-cancer therapy (Lord and Ashworth, 2012). Here, we summarize the role of ubiquitin-signaling in the DDR with special emphasis on its role in cancer and highlight the therapeutic value of the ubiquitin-conjugation machinery as a target in anti-cancer treatment strategy.

## Ubiquitin—small molecule generating a broad range of cellular actions

**Figure 1:**
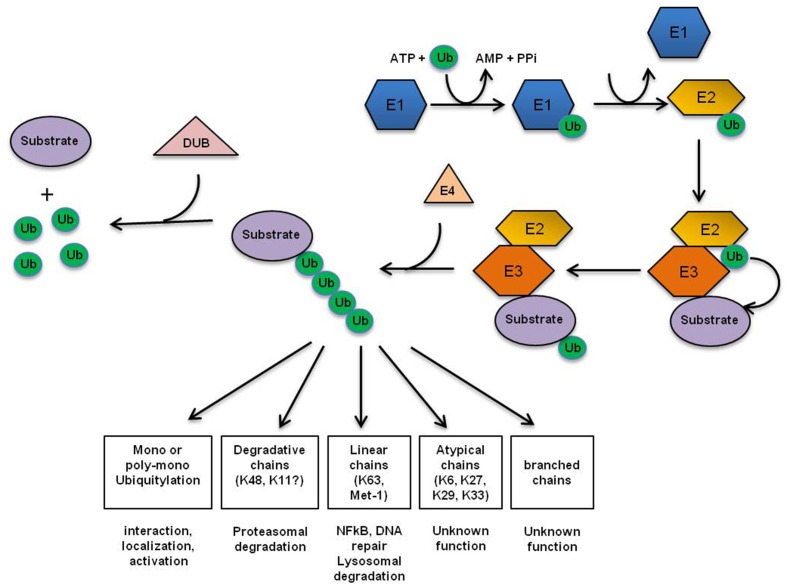
**Ubiquitin conjugation machinery**. Ub is attached to specific substrates in a three-step mechanism, with distinct enzymes catalyzing each step. First, Ub gets activated by the Ub-activating enzyme (E1). Next, activated Ub is transferred by one of several dozens of Ub-conjugating enzymes (E2) to one of approximately 500 substrate-specific Ub-ligases (E3s) that finally attaches Ub to the substrate (Pickart, 2001). In some cases, the extension of short ubiquitin chains requires additional elongation factors, termed E4 enzymes. About 100 substrate-specific deubiquitylating enzymes (DUBs) counteract the activity of UB-conjugating enzymes (Nijman et al., 2005). The first Ub is either transferred to a ε-NH2 group of a lysine residue (K) of the target protein to generate an isopeptide bond, or in a linear manner to the N-terminal residue of the substrate (Breitschopf et al., 1998; Pickart, 2001). Subsequent Ub addition can occur through isopeptide linkage on all of ubiquitin's seven lysine residues as well as its N-terminal primary amino group, thereby generating a diverse range of chain topologies (Met1-linked, K6, K11, K27, K29, K33, K48, K63 or mixed) that can drive a variety of different protein fates.

Ubiquitin (Ub) is an essential, highly conserved, 76 residue protein that is ubiquitously expressed in cells. It can be found either in a free form or covalently attached to a target protein (Schlesinger et al., 1975; Hershko et al., 1983; Ciechanover et al., 1985; Hershko and Ciechanover, 1998). Ub acts as a versatile cellular signal that controls a wide range of biological processes, including protein degradation, DNA repair, endocytosis, autophagy, transcription, immunity and inflammation. Ub, E1-, E2-, and E3-enzymes are successively required to target a certain substrate for degradation. Ub is attached to specific substrates in a three-step mechanism, with distinct enzymes catalyzing each step (Figure 1). In a first activating step, Ub becomes covalently conjugated to the side chain of an E1-cysteine via its carboxy-terminal (C-terminal) glycine in an ATP-dependent reaction. Activated Ub is then transferred to an E2-enzyme (ubiquitin-conjugating enzyme) via a thioester-bond between the C-terminal glycine residue of Ub and an E2 internal cysteine. Finally, Ub-bound E2 interacts with an E3 Ub ligase that catalyzes Ub transfer from E2 to a specific target protein (Ciechanover et al., 1984; Scheffner et al., 1995; Hershko and Ciechanover, 1998). This cascade of sequential interactions results in the formation of an isopeptide bond between the C-terminus of Ub and the ε-amino group of a lysine residue in the target protein (Hershko and Ciechanover, 1998). In some cases, the extension of short Ub chains requires additional elongation factors, called E4 enzymes. *Saccharomyces cerevisiae* ubiquitin fusion degradation 2 (*Ufd2)* is the first discovered E4 enzyme (Koegl et al., 1999; Hoppe, 2005). About 100 substrate-specific deubiquitylating enzymes (DUBs) counteract the activity of Ub-conjugating enzymes (Nijman et al., 2005). The specificity of Ub signaling is achieved by alternative conjugation signals (monoubiquitylation and more complex Ub chains) on alternative substrate sites (Haglund and Dikic, 2005). Diverse chain topologies can specify a variety of different protein fates by providing a platform for the interaction with specific binding partners. These interacting partners depend on Ub binding domains (UBD) or Ub interacting motifs (UIM) to either associate with Ub or to decode ubiquitylated target signals into biochemical cascades (Peng et al., 2003; Komander and Rape, 2012). For instance, monoubiquitylation plays a role in recognizing DNA double strand breaks (DSBs), K63-linked Ub chains are involved in the generation of signaling platforms during DNA repair (Chen et al., 2005a) and polyubiquitin chains covalently connected via K48 linkages mainly target proteins for degradation by the proteasome (Ciechanover et al., 1984; Thrower et al., 2000) (Figure 1). The ubiquitin/proteasome system (UPS) is one of the main regulators of protein stability and—among multiple cellular pathways—plays an important role in the execution of the DDR. Multiple studies using proteasome-inhibitors validated the UPS as a valuable therapeutic target in cancer (Voges et al., 1999; Orlowski and Kuhn, 2008); however, targeting one of the major cellular pathways governing protein turnover may cause broad and unspecific off-target cellular responses. Accordingly, ongoing efforts aim to identify the specific targets within the UPS system to selectively target the relevant Ub-conjugation process. Hence, novel Ub ligases or DUBs are frequently evaluated as potential specific targets for anti-cancer therapy.

## Recognition of DNA damage sites

Massive Ub accumulation around sites of DNA damage can be detected as soon as 15 s following the damage event (Feng and Chen, 2012). Ubiquitylation of the H2A, H2B, and H2AX histone subunits is one of the initial events promoting the destabilization of the nucleosome (Li et al., 1993; Biswas et al., 2011). CHFR (*checkpoint with Forkhead-associated (FHA) and RING finger domain protein*), which is recruited to DSBs by PAR (*poly (ADP) ribose*), regulates the first wave of histone ubiquitylation (Wu et al., 2011a). CHFR ubiquitylates PARP1 (*PAR polymerase 1*) via K48- (site K88, E2: UbcH5C) and K63-linked (E2: Ubc13) Ub chains, and this ubiquitylation is thought to promote the dissociation of PARP1 from damage sites (Liu et al., 2013). Epigenetic inactivation of *CHFR* has been described in several types of cancer, including breast cancer (Erson and Petty, 2004), nasopharyngeal carcinoma (Cheung et al., 2005), colorectal cancer (Toyota et al., 2003), head and neck cancer (Toyota et al., 2003), gastric cancer (Satoh et al., 2003), lung cancer (Mizuno et al., 2002), esophageal cancer (Shibata et al., 2002), hepatocellular cancer (Sakai et al., 2005) and T-cell lymphoma (van Doorn et al., 2005). Furthermore, increasing evidence indicate the regulatory impact of Ub on cancerogenesis. Monoubiquitylation of H2A by RNF2-BM1, a member of the Polycomb repressive complex 1 (PRC1), is thought to be important for the transcriptional repression by inhibiting of RNA-PolII-elongation (Zhou et al., 2008). Interestingly, around 15% of H2A has been described to be constitutively ubiquitylated (Levinger and Varshavsky, 1980). RNF2-BM1 is also involved in monoubiquitylation of H2AX at K119 and K120 (E2: UbcH5C), which in turn initiates the recruitment of the apical PI3K-related kinase *ataxia telangiectasia mutated* (ATM) (Pan et al., 2011; Wu et al., 2011a). ATM is a protein kinase that phosphorylates several key proteins involved in the DDR. So far, no role for this initial histone ubiquitylation in the recruitment of the functionally related apical kinases ATR (*ATM/Rad3-related kinase*) or DNA-PK (*DNA-dependent protein kinase*) has been demonstrated. ATM and ATR transduce the most upstream DDR signal by phosphorylating the checkpoint kinases CHK1/CHK2 and the tumor suppressor protein p53, resulting in cell cycle arrest to allow time for DNA repair, or DDIA after prolonged checkpoint activation, respectively (Shiloh, 2003). Even though the main function of DNA-PK appears to be the induction of cell cycle arrest and DNA repair, specifically the non-homologous end-joining (NHEJ) repair pathway, DNA-PK has also been reported to phosphorylate p53, thus cooperating with ATM/ATR to induce p53-mediated cell cycle arrest and apoptosis (Kim et al., 1999). Notably, the ubiquitin-selective segregase Cdc48/p97/VCP, which is a central regulator of the UPS, influences the DDR by participating in ubiquitylation and proteasomal degradation of the catalytic subunit of DNA-PK(cs) in eukaryotes (Acs et al., 2011; Meerang et al., 2011; Dantuma and Hoppe, 2012; Jiang et al., 2013). Consequently, the interplay of ubiquitylation and phosphorylation events regulates the association of several DDR proteins, most prominently p53, with regulatory E3 ligases or DUBs.

## p53—signal transducer from DNA damage to cellular actions

**Figure 2:**
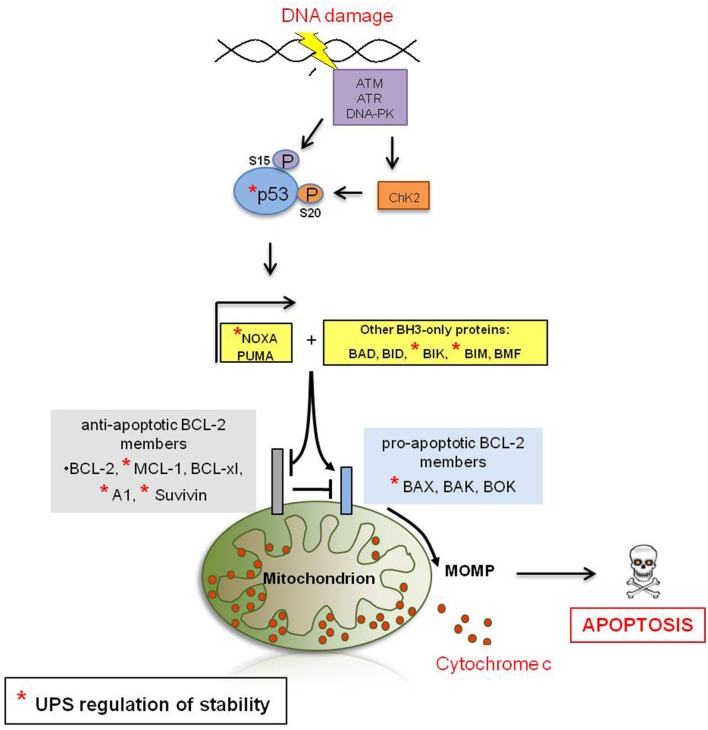
**DNA damage-induced apoptotic signaling**. The recruitment of ATM, ATR or DNA-PK to the site of DNA damage is a central event during DDR signaling. ATM and ATR transduce the DDR signal by phosphorylation of the checkpoint kinases CHK1/CHK2, which results in cell cycle arrest and either DNA repair or DDIA (Shiloh, 2003). Moreover, ATM and ATR are directly responsible for the post-translational stabilization and thus accumulation of the tumor supressor p53, a key player in transducing the DDR signal (see below in this figure). ATM directly phosphorylates p53 at residue S15 (Banin et al., 1998) and indirectly through the induction of the CHK2 kinase at residue S20 (Shiloh and Ziv, 2013). Phosphorylation of p53 is believed to be critical for the stabilization of p53. Activated p53 translocates into the nucleus where it induces the transcription of several targets involved in cell cycle regulation, DNA repair or apoptosis, including the pro-apoptotic molecule BAX (Miyashita and Reed, 1995) and the BH3-only proteins PUMA (Nakano and Vousden, 2001) and NOXA (Oda et al., 2000) which in turn induce MOMP either directly or in cooperation with other BH3-only proteins. Anti-apoptotic Bcl-2-family members inhibit apoptosis by antagonizing the induction of MOMP. Upon MOMP, multiple pro-apoptotic molecules are released from the mitochondrial intermembrane space (IMS) to activate aspartate proteases, called caspases, which ultimately coordinate most of the hallmarks of apoptosis and cellular self-destruction.

Activated p53 translocates into the nucleus where it induces the transcription of several target genes involved in cell cycle regulation, DNA repair, and apoptosis, including the pro-apoptotic molecule BAX (Miyashita and Reed, 1995) and the BH3-only proteins PUMA (Nakano and Vousden, 2001) and NOXA (Oda et al., 2000), which are central in initiating DDIA (Figure 2). Loss of p53 function is described in over 50% of human cancers and is frequently associated with a poor patient prognosis (Hollstein et al., 1994). The mechanisms by which p53 differentially triggers cell cycle arrest, senescence, and apoptosis are far from being completely understood; however, different post-translational modifications of p53 (e.g., phosphorylation) have been described that either alter its DNA binding capacity directly or that control its association with different binding partners, including transcriptional activators and repressors, thereby affecting the p53-induced transcriptome in response to DNA damage (Aylon and Oren, 2007). Moreover, it appears that p53 has different affinities toward different p53-responsive elements and different levels of p53 protein might fine-tune its promoter choice, thus determining cell fate. Indeed, low p53 levels tend to favor growth arrest, whereas higher levels trigger apoptosis (Laptenko and Prives, 2006). p53 protein stability is efficiently regulated by the UPS and several E3-ligases (Table 1) and DUBs (Table 2) have been reported as its direct regulators. Not surprisingly, most E3s or DUBs that regulate p53 stability are also implicated in cancer and further represent promising targets for anti-cancer therapy.

**Table 1:** **E3 ligases involved in DDIA**.

**E3 ligase**	**Target**
MDM2	MDMX (de Graaf et al., 2003; Kawai et al., 2003; Pan and Chen, 2003; Pereg et al., 2005; Xia et al., 2008), p53 (Momand et al., 1992)
COP1	p53 (Dornan et al., 2004a)
ARF-BP1/Mule	p53 (Chen et al., 2005b; Zhong et al., 2005; Parsons et al., 2009), ARF-BP1/Mule (Chen et al., 2005b), MCL-1 (Zhong et al., 2005)
PIRH2	p53 (Sheng et al., 2008), CHK2 (Bohgaki et al., 2013), PIRH2 (Logan et al., 2004; Abou Zeinab et al., 2013), p73 (Jung et al., 2011; Wu et al., 2011c)
Cul4B	p53 (Nag et al., 2004; Thirunavukarasou et al., 2014)
E6-AP	p53 (Scheffner et al., 1993)
Cul4A-DDB1	p53 (Nag et al., 2004), p73 (Malatesta et al., 2013)
ITCH	p63 (Rossi et al., 2006), p73 (Rossi et al., 2005), tBID (Azakir et al., 2010)
SCF^Fbw7^	MCL-1 (Inuzuka et al., 2011; Wertz et al., 2011)
SCF^βTrCP^	MCL-1 (Ding et al., 2007), BIM (Dehan et al., 2009)
APC/Cdc20	MCL-1 (Harley et al., 2010)
TRIM17	MCL-1 (Magiera et al., 2013)
SAG/RBX2	BIM (Li et al., 2014)
TRIM2	BIM (Thompson et al., 2011)
Culin/ElonginB-CIS	BIM (Ambrosini et al., 2009)
RNF186	BNip1 (Wang et al., 2013)

**Table 2:** **DUBs involved in DDIA**.

**DUB**	**Target**
USP7	p53 (Li et al., 2002), ARF-BP-1/Mule (Khoronenkova and Dianov, 2013)
USP4	ARF-BP-1/Mule (Zhang et al., 2011)
USP2a	MDM2 (Stevenson et al., 2007), MDMX (Allende-Vega et al., 2010)
USP10	p53 (Yuan et al., 2010)
USP42	p53 (Hock et al., 2011)
USP29	p53 (Liu et al., 2011)
UCH-L1	^*^p53 (Li et al., 2010; Xiang et al., 2012), NOXA (Brinkmann et al., 2013)
Otubain 1	^*^p53 (Sun et al., 2012)
USP9X	MCL-1 (Schwickart et al., 2010)
USP18	^*^BIM (Santin et al., 2012)

* Indirect stabilization, no direct deubiquitylation reported.

### Regulation of p53 by ubiquitin ligases

The E3 ligase MDM2 has been shown to directly target p53 for proteasomal degradation while ATM/ATR-mediated phosphorylation of p53 hampers this interaction (Momand et al., 1992); however, MDM2 only mediates monoubiquitylation of p53, but not its polyubiquitylation, arguing for the involvement of additional Ub ligases (Lai et al., 2001). Interestingly, MDM2 is a transcriptional target of p53 and thus acts in a negative feedback loop. Furthermore, MDM2 itself is also a target of ATM and ATM-dependent phosphorylation of MDM2 precedes p53 accumulation in response to DNA damage (Khosravi et al., 1999). ATM-mediated phosphorylation of MDM2 at S395 induces MDM2 protein destabilization. One major molecule that has been further implicated in regulating MDM2-mediated p53 proteolysis is MDMX (MDM4). MDMX activity seems to be essential for MDM2-mediated p53 proteolysis by converting MDM2 into an active conformation (Di Conza et al., 2012) and further stimulating MDM2 ligase activity (Linke et al., 2008; Wang et al., 2011; Wade et al., 2012). ATM-mediated MDM2/MDMX phosphorylation disrupts MDM2 oligomerization, thereby inactivating MDM2, leading to p53 stabilization and activation (Cheng and Chen, 2010); however, MDM2 additionally promotes ubiquitylation and degradation of MDMX in response to DNA damage, again acting in a negative feedback loop (de Graaf et al., 2003; Kawai et al., 2003; Pan and Chen, 2003; Pereg et al., 2005; Xia et al., 2008). Additional studies showed that MDM2 generates non-degradative polyubiquitin chains indicating an additional function of MDM2/MDMX-ubiquitylation other than the previously demonstrated degradative target ubiquitylation (Badciong and Haas, 2002). An additional factor in the negative feedback loop regulating p53 is the phosphatase Wip1. Wip1 acts as a gatekeeper in this regulatory loop by dephosphorylating and thus stabilizing MDM2, promoting MDM2-mediated p53 proteolysis (Lu et al., 2007). Elevated expression of negative regulators of p53-stability is reported for numerous tumors and is strongly associated with a poor patient prognosis. For instance, elevated expression of MDM2 has been identified in breast cancer (Bueso-Ramos et al., 1996), leukemias (Bueso-Ramos et al., 1993), and in lung cancer (Dworakowska et al., 2004). Gene amplifications of MDM2 have been described in 7% of tumors of diverse origin, with the highest frequency observed in soft tissue tumors, osteosarcomas and esophageal carcinomas (Momand et al., 1998). MDM2 is also a substrate for alternative splicing and the production of aberrantly spliced MDM2 RNA is associated with a shortened overall survival of cancer patients (Bartel et al., 2002). Remarkably, a functional interaction of p53/MDM2 is dispensable for embryonic development, whereas it is essential for DDIA, thus emphasizing the potential of the p53/MDM2-interaction as a target in anti-cancer therapy (Zhao et al., 2013).

The E3 ligase COP1 regulates p53 stability in an ATM-dependent manner (Dornan et al., 2004a). Upon DNA damage, activated ATM phosphorylates COP1 on S387 which in turn stimulates a rapid autodegradation mechanism of COP1 resulting in p53 stabilization (Dornan et al., 2006). COP1 itself is a transcriptional target of p53, thus representing yet another feedback loop for controlling p53 stability (Dornan et al., 2004a). Overexpression of *COP1* has been observed in breast cancer (Dornan et al., 2004b), ovarian adenocarcinoma (Dornan et al., 2004b), gastric cancer (Li et al., 2012; Sawada et al., 2013), and in hepatocellular carcinoma (Lee et al., 2010) and this high expression is mostly associated with a poor prognosis.

p53 stability is also negatively controlled by ARF-BP1/Mule encoded by the *Huwe1* gene, which is a binding partner of the alternative binding frame (ARF) tumor suppressor (Chen et al., 2005b; Zhong et al., 2005; Parsons et al., 2009). ARF-BP-1/Mule activity is limited by self-ubiquitylation and subsequent proteasomal turnover (Chen et al., 2005b). Increased ARF-BP1/Mule degradation causes p53 stabilization, which is antagonized by the DUBs USP7 (Khoronenkova and Dianov, 2013) and USP4 (Zhang et al., 2011). ARF-BP1/Mule was found to be expressed at high levels in lymphoma cell lines (Qi et al., 2012) and in colorectal and breast cancer cells (Xie et al., 2002) and it promotes Myc-driven tumorigenesis (Qi et al., 2012), whereas it suppresses Ras-driven tumorigenesis (Inoue et al., 2013).

Another specific E3 ligase for p53 is PIRH2, which was initially named p27(Kip1) and implicated in cell cycle regulation (Leng et al., 2003). Remarkably, PIRH2 preferentially ubiquitylates the transcriptional active form of p53 (Sheng et al., 2008). Moreover, PIRH2 also regulates the stability of the effector kinase CHK2 (Bohgaki et al., 2013) and phosphorylation of PIRH2 by calmodulin-dependend kinase 2 impairs its ability to ubiquitylate p53 (Duan et al., 2007). Again, PIRH2 levels are regulated by self-ubiquitylation following proteasomal turnover (Logan et al., 2004; Abou Zeinab et al., 2013). *PIRH2* is overexpressed in a variety of tumor cells including hepatocellular carcinoma (Wang et al., 2009; Hu et al., 2012), head and neck cancers (Shimada et al., 2009), clear renal cell carcinoma (Wu et al., 2013), lung cancer (Duan et al., 2004), and prostate cancer (Logan et al., 2006).

A number of additional E3 ligases are reported to regulate p53 degradation, including Cul4B (Thirunavukarasou et al., 2014), E6-AP (Scheffner et al., 1993), and Cul4A-DDB1 (Nag et al., 2004; Thirunavukarasou et al., 2014). Strikingly, regulation of p53 protein is also influenced by the activities of the E4 ligases UBE4B/UFD2a/Ufd2 (Wu et al., 2011b) and CBP (CREB-binding protein)/p300 (E1A binding protein p300) (Shi et al., 2009).

E4 ligases mediate the polyubiquitylation of specific monoubiquitylated substrate proteins, including p53. Recently, CBP and p300 were identified to possess E4 activity and can elongate monoubiquitylated p53 into the cytosolic polyubiquitylated form (Shi et al., 2009). In addition, the E4 ligase UBE4B interacts physically with p53 and MDM2 to polyubiquitylate p53 (Wu et al., 2011b). Consequently, elevated levels of UBE4B are linked to brain tumors and medulloblastoma cell lines. It was further observed that the gene locus of *UBE4B* (1p36.22) is a susceptible candidate locus for hepatitis B virus (HBV) related hepatocellular carcinoma (HCC), forming a possible link between UBE4B/UFD2 and cancer development and tumor suppression (Zhang et al., 2010; Wu and Leng, 2011; Wu et al., 2011b).

### Regulation of p53 by DUBs

So far, several DUBs are known to regulate p53 stability, either directly by deubiquitylation and stabilization of p53 itself, or by regulating its key regulators or binding partners. The ubiquitin-specific protease USP7 (HAUSP—herpes virus associated USP) was initially found to be a specific DUB of p53 and its activity stabilizes p53 protein (Li et al., 2002). However, whereas decreased USP7 expression levels had the expected effect of destabilizing p53, ablation of USP7 expression was found to have the opposite effect, resulting in p53 stabilization (Sheng et al., 2006). This p53 stabilization seems to result from increased ubiquitylation and destabilization of MDM2, the E3 ligase largely responsible for p53 ubiquitylation (Cummins and Vogelstein, 2004; Li et al., 2004; Meulmeester et al., 2005). USP2a has been described as a specific DUB of MDM2 (Stevenson et al., 2007) and MDMX (Allende-Vega et al., 2010) and thereby acts as a negative regulator of p53 stability. USP10 is a cytosolic DUB that specifically deubiquitylates p53, while ATM-mediated phosphorylation results in USP10 stabilization as well as nuclear translocation, resulting in p53 stabilization (Yuan et al., 2010). USP42 and USP29 are DUBs for p53 and improve p53 stability under stress conditions (Hock et al., 2011; Liu et al., 2011). Similarly, positive regulation of p53 stability has also been described for OTUB1 (Otubain1), which stabilizes p53 indirectly and independently of its catalytic activity by binding the E3 ligase MDM2. This interaction inhibits the cooperation between MDM2 and UbcH5s, the E2 enzyme important for MDM2-mediated p53 ubiquitylation (Sun et al., 2012). UCH-L1 has also been reported to regulate p53 protein stability (Li et al., 2010; Xiang et al., 2012); however, the molecular details are yet not clear.

Examples for DUBs that might antagonize E4 dependent polyubiquitylation are USP47, a regulator of Base Excision Repair (BER) that controls DNA polymerase β and OTUB1, which mediates DNA damage-dependent deubiquitylation of p53/MDM2 in the cytoplasm (Parsons et al., 2011; Sun et al., 2012).

### Regulation of the p53 homologs p63 and p73 by ubiquitin ligases

Interestingly, p53 is required for the DDR in certain but not all cell types (Clarke et al., 1993; Lowe et al., 1993; Strasser et al., 1994). Even though the primary role is exerted by p53 itself, the p53 homologs p63 and p73 can substitute for the downstream activities of p53. p63 and p73 share 60% similarity with the p53 DNA binding domain, allowing them to transactivate some of the same target genes. Like p53, p73 proteasomal turnover is regulated by the E3 ligase PIRH2 (Jung et al., 2011; Wu et al., 2011c) and also by the E4 ligase UFD2a (Hosoda et al., 2005). Furthermore, p63 and p73 protein stability are directly regulated by the ubiquitin ligase ITCH (Rossi et al., 2005, 2006). p73 is a substrate of the Cul4A-DDB1 Ub ligase complex which monoubiquitylates p73 thereby reducing its transcriptional activity without affecting its turnover (Malatesta et al., 2013). MDM2 also binds p73 without supporting its degradation (Balint et al., 1999). Likewise, MDMX, but not MDM2, has been shown to regulate p63 transactivation potential by inhibiting p63 nuclear localization (Kadakia et al., 2001).

## DNA repair mechanisms

DSB repair is mediated by two extensively studied major repair pathways that have evolved in eukaryotic cells (Chapman et al., 2012). The error prone NHEJ pathway reunites free DNA ends at DSBs with little or no sequence homology and is responsible for most of the repair events in eukaryotes (Lemmens and Tijsterman, 2010). Repair via NHEJ can be rather inexact because the rejoining of non-complementary DNA ends is subject to end-processing by the nuclease activity of Artemis and DNA-PK(cs), which remove damaged or mismatched nucleotides (Bunting and Nussenzweig, 2013). Accurate ligation depends on the presence of loose complementary cohesive DNA ends and is mediated by the NHEJ repair proteins Ku70/80 and XRCC4-Ligase IV (Dahm-Daphi et al., 2005; Moynahan and Jasin, 2010). A second repair pathway is homologous recombination (HR), which dominates in highly proliferative somatic cells in S- and G2-phase. HR is a high fidelity repair pathway that relies on recombination between undamaged sister chromatids or homologous chromosomes (Clejan et al., 2006). Ubiquitylation of substrate proteins plays an important role in specifying the use of a specific DNA repair pathway, as differential ubiquitylation leads to orchestrated recruitment of specific repair factors such as p53-binding protein 1 (53BP1) or Breast Cancer Susceptibility Gene 1 (BRCA1) (Jackson and Durocher, 2013). 53BP1 accumulation promotes NHEJ activation and HR inhibition, whereas BRCA1 recruitment triggers HR (Yun and Hiom, 2009). Their recruitment to chromatin surrounding DSB sites is controlled by the action of the RING-finger protein RNF8, which acts as a central E3 ligase in DDR and exhibits two distinct roles: it catalyzes the ubiquitylation of substrate proteins either via a protein-recruiting K63- or via a destabilizing K48 specific linkage (Lok et al., 2012). Upon DNA damage RNF8 detects motifs in mediator of DNA damage checkpoint protein 1 (MDC1) previously phosphorylated by ATM and performs K63-linked monoubiquitylation of histones H2A and H2AX. Histone monoubiquitylation promotes RNF8-dependent recruitment of a second E3 ligase, RNF168, to the damage site, which can identify ubiquitylated RNF8 substrates via its Nterminal ubiquitin-binding domains (Mailand et al., 2007; Doil et al., 2009). Subsequently, polyubiquitylation of H2AX further promotes the recruitment of RNF168 to the damage site, amplifying RNF8-dependent histone ubiquitylation by ubiquitylating other substrate proteins via K63 (Doil et al., 2009; Ramadan and Meerang, 2011). The outcome of RNF8/RNF168-dependent K63-linked ubiquitylation is the generation of a molecular landing platform for the accumulation of checkpoint and DNA repair proteins like BRCA1 or 53BP1; however, 53BP1 itself cannot directly bind to K63 polyubiquitin chains since it lacks any relevant binding site (Al-Hakim et al., 2010). Therefore, other mechanisms for 53BP1 recruitment are necessary. For example, 53BP1 accumulation is promoted by p97 segregase activity that removes the polycomb protein L3MBTL1 from DNA DSBs. p97 binds to ubiquitylated L3MBTL1 and extracts it from chromatin. The displacement of L3MBTL1 unmasks 53BP1 binding sites that can now be occupied (Acs et al., 2011).

In addition, RNF8 also ubiquitylates K48-dependent substrates such as the lysine demethylase JMJD2A (Mallette et al., 2012), the NHEJ repair protein Ku80 (Feng and Chen, 2012), and the DNA polymerase sliding clamp proliferating cell nuclear antigen (PCNA), which is involved in DNA synthesis and repair (Zhang et al., 2008). Consequently, these proteins are removed from chromatin for proteasomal degradation.

In accordance with the postulated molecular switch model of PCNA, E4-mediated polyubiquitylation might alter ubiquitin-dependent signaling fates upon damage induction, possibly in a cell type specific manner (Hoppe, 2005). This regulatory mechanism thereby provides another layer of regulation to fine-tune the highly dynamic cascade of ubiquitylation events during the DDR, which can also be reversed by DUB activity.

Besides K48-linked ubiquitylation, PCNA undergoes a switch mechanism from a mono- to a polyubiquitylated form at position K164, regulating its activity in DNA repair (Hoege et al., 2002). This modification triggers translesion synthesis (TLS), i.e., DNA synthesis across lesions. In addition, other factors are needed to extend the modification by a K63-linked polyubiquitin chain leading to an error-free pathway of damage avoidance (Hoege et al., 2002; Daigaku et al., 2010).

A different ubiquitin chain linkage was reported for the E3 ubiquitin ligase BRCA1, which exhibits tumor-suppressor activities and is crucial for maintaining genomic integrity. As a heterodimer with its binding partner BARD1 it specifically catalyzes the formation of K6-linked polyubiquitin chains on substrates, such as RNA Polymerase II and γ-Tubulin (Wu-Baer et al., 2003; Irminger-Finger and Jefford, 2006).

## DDIA

In addition to the activation of DNA repair, multicellular organisms acquired a dynamic safe-guard system involving the apoptotic response to dispose of damaged cells when the extent of damage is beyond the cellular repair capacity (Levine et al., 1997). The decision whether a cell survives or dies upon DNA damage is not yet completely understood, however, as mentioned above, the level of p53 abundance is a key factor in the cellular decision of life or death in response to DNA damage. Similarly, the quality of p53 downstream death signaling—the induction of intrinsic/mitochondrial apoptosis—plays a crucial role in the coordinated cellular death upon DNA damage. Specifically, the expression level of pro- and anti-apoptotic proteins, in particular, members of the Bcl-2-family (see below), is decisive for the outcome of the DDR signaling. Furthermore, the nature of the DNA damage, the physiologic status and the origin of the damaged cell may impact on cellular responses to DNA damage. For instance, thymocytes are highly primed to undergo DDIA, whereas primary fibroblasts appear to resist DDIA (Norbury and Zhivotovsky, 2004). Indeed, the capability of the apoptotic machinery in immune cells is central during the cellular differentiation of this tissue. For instance, almost 90% of pre-T- and B-cells undergo apoptosis during maturation. Further, apoptosis triggers the shutdown of the immune response when infection has been overcome (Brinkmann and Kashkar, 2014). In conclusion, several cell types are primed for a rapid induction of apoptosis which is achieved by a “close-to-death” composition of pro- and anti-apoptotic proteins, in particular, Bcl-2-family members (Letai et al., 2002).

Nevertheless, a tight regulation of the response to DNA damage is obligatory in germ cells and somatic cells. In germ cells, mechanisms for limiting genome alterations are required for faithful propagation of the species, whereas in somatic cells, responses to DNA damage prevent the accumulation of mutations that might lead to altered cellular homeostasis.

### Bcl-2 protein family—regulators of mitochondrial apoptosis

Mitochondria represent a central regulatory node in the apoptotic machinery through the mitochondrial outer membrane permeabilization (MOMP) as the decisive event. Upon MOMP, multiple pro-apoptotic molecules, including cytochrome C are released from the mitochondrial intermembrane space (IMS) to activate aspartate proteases, called caspases, which ultimately coordinate most of the hallmarks of apoptosis and cellular self-destruction. Specifically, cytosolic cytochrome C forms a complex, the apoptosome, with ATP, APAF1, and pro-caspase 9 (pro-casp9), resulting in the activation of caspase 9 (casp9). Casp9 activates the downstream executioner caspase 3 (casp3) which ultimately lead to apoptosis.

Inefficient MOMP has been suggested to be one of the key determinants of therapeutic success of a number of anti-cancer regimens in cancer patients (Adams and Cory, 2007) and members of the Bcl-2 protein family are the key-regulators of this process. The Bcl-2 protein family comprises three classes of member. The first group consists of the anti-apoptotic Bcl-2 protein family members, including BCL-2, BCL-xl, BCL-w, A1, and MCL-1, that efficiently inhibit MOMP and block apoptosis. The second group consists of pro-apoptotic members such as BAK, BAX, and BOK, trigger apoptosis by directly promoting MOMP. A third divergent class of BH3-only proteins including BIM, BID, PUMA, BAD, and NOXA regulates the activity of pro-and anti-apoptotic Bcl-2 proteins (Adams and Cory, 2007) (Figure 2).

Members of the Bcl-2 protein family share at least one conserved Bcl-2 homology domain (BH domain), which is characterized by several α-helical segments. The BH domain does not possess enzymatic activity but it allows pro- and anti-apoptotic members to bind to and to inhibit each other (Adams and Cory, 1998; Cory and Adams, 2002). Binding affinity assays using BH3-only peptides revealed that not all pro- and anti-apoptotic Bcl-2 proteins can antagonize each other, but the affinity differs within the family. The BH3-only proteins BIM, BID, PUMA, and BMF can bind and antagonize all anti-apoptotic Bcl-2 proteins. In contrast, BAD can only bind BCL-2, BCL-xl and BCL-w, and NOXA is restricted in binding to MCL-1 and A1. To date, there are two proposed models that explain how the Bcl-2 protein family regulates MOMP: (i) the indirect activator model and (ii) the direct activator-derepressor model. Both models result in the activation of BAX and BAK and the permeabilization of the outer mitochondrial membrane. The indirect activator model postulates that BAX and BAK are bound in a constitutively active state to anti-apoptotic Bcl-2 proteins. Competitive interactions with pro-apoptotic BH3-only proteins and anti-apoptotic Bcl-2 proteins are sufficient to release active BAX and BAK and induce MOMP. In the direct activator-derepressor model (also called neutralization model), BAX and BAK are activated by the interaction with a subset of BH3-only proteins, such as BID and BIM, called direct activators. In this model, anti-apoptotic Bcl-2 proteins either inhibit MOMP by antagonizing BAX or BAK directly or by sequestering the direct activator BH3-only proteins, thus preventing them from activating BAX or BAK. A second subset of BH3-only proteins, called sensitizers, such as NOXA or BAD, cannot directly activate BAX or BAK but antagonize anti-apoptotic Bcl-2 proteins and thereby release BAX and BAK for the activation by direct activator BH3-only proteins (Tait and Green, 2010).

In response to DNA damage activated p53 translocates into the nucleus where it induces transcription of several pro-apoptotic Bcl-2 proteins, including BAX (Miyashita and Reed, 1995), PUMA (Nakano and Vousden, 2001), and NOXA (Oda et al., 2000), which in turn induce MOMP. The transcriptional upregulation of these pro-apoptotic members in response to DNA damage however may not suffice the required pro-apoptotic trigger toward MOMP as this process is tightly regulated by a number of other Bcl-2 members and only the ultimate pro-apoptotic composition of these proteins can efficiently induce cell death (Ni Chonghaile and Letai, 2008). Accordingly, the genes of some BH3-only proteins appear to be constitutively transcribed in cancer cells as reported for BIK or NOXA (Hur et al., 2004; Brinkmann et al., 2013; Dengler et al., 2014). The majority of these cells however resist apoptosis suggesting that the imbalance in Bcl-2 protein family members (e.g., upregulation of anti-apoptotic members or downregulation of BAX/BAK) efficiently counter the pro-apoptotic action of these factors. More strikingly, non-transcriptional regulation of Bcl-2 protein family members turn-over was repeatedly shown to control the apoptotic process under physiological or pathological condition. This enables cells to rapidly respond to stress cues by regulating protein abundance without employing protein *de novo* synthesis.

The clinical successes of proteasome inhibitors for the treatment of cancer have highlighted the therapeutic potential of targeting cellular process governing protein turn-over. Strikingly, the expression levels of a number of Bcl-2 protein family members including NOXA (Qin et al., 2005; Brinkmann et al., 2013), MCL-1 (Adams and Cory, 2007), A1 (Kucharczak et al., 2005), BCL-2 (Dimmeler et al., 1999), BAK (Qin et al., 2005), BIK (Marshansky et al., 2001; Hur et al., 2004), BIM (Nikrad et al., 2005) was altered when the proteasome was inhibited indicating an essential role of the UPS in regulating Bcl-2-family protein abundance. However, a direct regulation of Bcl-2-protein level via the UPS has only been reported for BAX (Chang et al., 1998; Li and Dou, 2000), BIM (Akiyama et al., 2003), BCL-2 (Dornan et al., 2004b), NOXA (Brinkmann et al., 2013), MCL-1 (Zhong et al., 2005), A1 (Kucharczak et al., 2005), and BCL-B (van de Kooij et al., 2013), while the identities of the responsible E3 ligases and DUBs are largely unknown with some exceptions (Tables 1, 2).

Previous data showed that the stability of the anti-apoptotic BCL-2 protein is regulated through ubiquitylation which is in turn controlled by its phosphorylation (Breitschopf et al., 2000a; Basu and Haldar, 2002). Specifically, MAP kinase-mediated BCL-2 phosphorylation was shown to block BCL-2 ubiquitylation and proteasomal degradation (Dimmeler et al., 1999). Furthermore, BCL-2 turn-over is inhibited by its direct S-nitrosylation (Azad et al., 2006; Chanvorachote et al., 2006). These data showed that BCL-2 undergoes S-nitrosylation by endogenous nitric oxide (NO) in response to multiple apoptotic stimuli. S-nitrosylation of BCL-2 in turn inhibits its proteasomal degradation.

The level of MCL-1 protein is regulated by the action of at least five distinct E3-ligases, namely ARF-BP1/Mule (Zhong et al., 2005), SCF^Fbw7^ (Inuzuka et al., 2011; Wertz et al., 2011), SCF^βTrCP^ (Ding et al., 2007), APC/Cdc20 (Harley et al., 2010), Trim17 (Magiera et al., 2013), and the DUB USP9X (Schwickart et al., 2010). Whether different E3-ligases are engaged in different cellular action and in response to different stimuli is not determined. Independently, ubiquitylation of MCL-1 has been mainly considered as a regulatory circuit controlling its abundance. Not surprisingly, dysregulation of MCL-1 ubiquitylation and turn-over have been repeatedly associated with cancer and cancer chemoresistance (Schwickart et al., 2010; Wertz et al., 2011). Mule-dependent MCL-1 ubiquitylation is enhanced by NOXA, which targets Mule to MCL-1 and competes with USP9X in MCL-1 binding (Gomez-Bougie et al., 2011). These data suggest that NOXA, in addition to the functional antagonization of MCL-1, controls MCL-1 turn-over by regulating the physical interaction of MCL-1 with ubiquitin conjugation/deconjugation machinery.

Independent of its own inherent pro-apoptotic activity, the critical role of NOXA in regulating MCL-1 is a unique property of this protein among other BH3-only protein family members. NOXA was initially identified as a primary p53-responsive gene, providing the first evidence for the transcriptional regulation of NOXA in response to genotoxic stress (Oda et al., 2000). In addition to transcriptional regulation, NOXA stability is controlled by post-translational mechanisms. In particular, ubiquitylation of NOXA has recently been shown to be involved in the regulation of NOXA protein turn-over and thereby influences cellular stress responses (Baou et al., 2010; Dengler et al., 2014). Interfering with this process, dysregulation of NOXA ubiquitylation has been shown to be an efficient strategy of some tumor cells in order to resist the genotoxic chemotherapy (Brinkmann et al., 2013). Specifically, these data showed that NOXA was strongly ubiquitylated in some tumor samples. The elevated NOXA ubiquitylation and reduced stability was a result of epigenetic silencing of NOXA-specific DUB, UCH-L1, which directly deubiquitylates and stabilizes NOXA (Brinkmann et al., 2013). Furthermore, NOXA can be degraded by an ubiquitin-independent mechanism suggesting that the disruption of 26S proteasome function by various mechanisms triggers the rapid accumulation of NOXA based on the capability of NOXA to act as a sensor of 26S proteasome integrity (Craxton et al., 2012).

Ubiquitin-dependent degradation of BIM is regulated by the E3 ligases SAG/RBX2 (Li et al., 2014), TRIM2 (Thompson et al., 2011), Cullin/ElonginB-CIS (Ambrosini et al., 2009), and SCF^βTrCP^ (Dehan et al., 2009) while phosphorylation through different kinases including ERK1/2, MAPK and the cell cycle kinase Aurora A precedes its turnover (Ley et al., 2003; Ramesh et al., 2008; Dehan et al., 2009; Wiggins et al., 2010). USP18 has also been shown to be involved in regulating the stability of BIM upon cytokine-induced cell death. Specifically, USP18 inhibition in INS-1E cells enhanced BIM expression level in untreated and IFNγ-treated conditions (Santin et al., 2012).

Analysis of BAX stability in human prostate adenocarcinoma showed that BAX is highly instable and the reduced BAX protein levels was associated with increased Gleason scores of prostate cancer (Chang et al., 1998; Li and Dou, 2000). These results identified the UPS-mediated BAX degradation as a novel survival mechanism in tumor cells and suggested that a selective targeting of this pathway should provide a unique approach for treatment of human cancers, especially those overexpressing BCL-2 (Chang et al., 1998; Li and Dou, 2000).

The stability of the BH3-only protein BNip1 is regulated via the action of the E3 ligase RNF186. BNip1 co-localizes with RNF186 at the ER and is poly-ubiquitylated by RNF186 through K29 and K63 linkage *in vivo*. This modification promotes BNip1 transportation to mitochondria but has no influence on its protein level (Wang et al., 2013).

Extrinsic apoptotic cascade results in the proteolytic activation of BID by caspase-8 (Luo et al., 1998). The COOH-terminal cleavage fragment of BID (truncated BID, tBID) becomes localized to mitochondrial membranes and triggers the release of cytochrome c. Truncated BID was shown to be ubiquitylated and subsequently degraded by the 26 s proteasome which is believed to control the extent of apoptosis in living cells (Breitschopf et al., 2000b). Further analyses identified the ubiquitin ligase ITCH, as a specific ubiquitin ligase of tBID which was not able to use intact BID as a substrate and initiate its proteasomal degradation (Azakir et al., 2010). The N-terminal cleavage product of BID has also been shown to be a substrate of unconventional ubiquitylation and degradation as the acceptor site are neither lysines nor N-terminus (Tait et al., 2007). Acceptor sites reside predominantly but not exclusively in helix 1, which is required for ubiquitylation and degradation of tBID-N. Rescue of tBID-N from degradation blocked BID's ability to induce mitochondrial outer membrane permeability but not mitochondrial translocation of the cleaved complex.

The increasing number of ubiquitin-conjugation events, regulating the abundance or function of Bcl-2 protein family members, is a strong indication of the central role of Ub in DDIA and provides at the same time a promising therapeutic target for cancer treatment.

## Exploiting ubiquitin-signaling in DDR as a therapeutic target in cancer

The ultimate central goal of conventional cancer therapy is the effective elimination of tumors by invoking DDIA. Since the balance of protein abundance and functionality are decisive for DDR outcomes, it is not surprising that deregulation of ubiquitin-signaling pathways is intimately associated with tumorigenesis and therapy resistance. Accumulating recent evidence conclusively identified ubiquitin-signaling as a valuable target in DDR and cancer chemoresistance. The majority of these efforts focused on the regulation of p53 as one of the central determinants of DDR outcomes. Accordingly, an increasing number of specific regulators of p53 have been identified and evaluated as therapeutic targets. RITA (reactivation of p53 and induction of tumor cell apoptosis) is a small molecule that blocks p53/MDM2 interaction (Issaeva et al., 2004); however, it appeared to be rather unspecific since its pro-apoptotic capacity was described to be p53-independent in several tumors, including myelomas (Surget et al., 2014) and additional data indicated that RITA cannot inhibit this interaction *in vitro* (Krajewski et al., 2005). Nutlins are also described to block the interaction of p53 and MDM2 (Vassilev, 2004). These molecules activate the p53 pathway and suppress tumor growth *in vitro* and *in vivo* in tumor xenograft models of solid and hematologic tumors (Vassilev et al., 2004; Tovar et al., 2006; Sarek and Ojala, 2007). MI-63 and MI-219 are small molecules also designed to block the interaction between p53/MDM2 and early preclinical evaluations demonstrated p53-mediated cell cycle arrest or apoptosis in tumor xenograft models upon treatment with Mi-219 (Shangary et al., 2008). P28 is a peptide fragment derived from azurin, a redox protein secreted from *Pseudomonas aeruginosa*, which stabilizes p53 by blocking its interaction with COP1 (Yamada et al., 2013a,b). The first preclinical trials demonstrated inhibition of tumor growth in xenograft models of p53 positive solid tumors (Jia et al., 2011).

## Conclusion

Tumor cell resistance to genotoxic chemotherapy poses a significant challenge in the treatment of cancer patients. As already discussed, protein ubiquitylation is central to the orchestration of the DDR and impacts on susceptibility to conventional genotoxic chemotherapy. Recent studies using proteasome-inhibitors validated the UPS as a therapeutic target in cancer and provided an impetus to promote the development of effective novel drugs that more specifically interfere with the ubiquitin-conjugating machinery. Thus, a better understanding of the specific link between the DDR and the ubiquitin-conjugating machinery will undoubtedly identify novel targets involved in cancer and will promote the development of new therapeutic strategies to overcome cancer chemoresistance. In line with this notion, based on its ability to inhibit apoptosis, the Bcl-2 protein family has garnered the most attention as a promising therapeutic target in cancer. Accordingly, efforts have lately been focused on the development of drugs targeting Bcl-2 proteins with considerable therapeutic success (Brinkmann and Kashkar, 2014). In view of the fact that the acquired imbalance of Bcl-2 proteins is involved in cancer together with our increasing knowledge about the central role of ubiquitin-conjugation governing Bcl-2 abundance and function support the idea that cancer-treatment may strongly benefit from novel therapeutic protocols targeting ubiquitin-regulation of Bcl-2 family.

### Conflict of interest statement

The authors declare that the research was conducted in the absence of any commercial or financial relationships that could be construed as a potential conflict of interest.
